# Fenton-Chemistry-Based Oxidative Modification of Proteins Reflects Their Conformation

**DOI:** 10.3390/ijms22189927

**Published:** 2021-09-14

**Authors:** Thomas Nehls, Tim Heymann, Christian Meyners, Felix Hausch, Frederik Lermyte

**Affiliations:** Clemens-Schöpf-Institute, Department of Chemistry, Technical University of Darmstadt, Alarich-Weiss-Straße 4, 64287 Darmstadt, Germany; thomas.nehls@tu-darmstadt.de (T.N.); tim.heymann@tu-darmstadt.de (T.H.); christian_stephan.meyners@tu-darmstadt.de (C.M.); felix.hausch@tu-darmstadt.de (F.H.)

**Keywords:** mass spectrometry, protein folding, protein–ligand interactions, protein dynamics, FK506-binding protein, FKBP12, FKBP51

## Abstract

In order to understand protein structure to a sufficient extent for, e.g., drug discovery, no single technique can provide satisfactory information on both the lowest-energy conformation and on dynamic changes over time (the ‘four-dimensional’ protein structure). Instead, a combination of complementary techniques is required. Mass spectrometry methods have shown promise in addressing protein dynamics, but often rely on the use of high-end commercial or custom instruments. Here, we apply well-established chemistry to conformation-sensitive oxidative protein labelling on a timescale of a few seconds, followed by analysis through a routine protein analysis workflow. For a set of model proteins, we show that site selectivity of labelling can indeed be rationalised in terms of known structural information, and that conformational changes induced by ligand binding are reflected in the modification pattern. In addition to conventional bottom-up analysis, further insights are obtained from intact mass measurement and native mass spectrometry. We believe that this method will provide a valuable and robust addition to the ‘toolbox’ of mass spectrometry researchers studying higher-order protein structure.

## 1. Introduction

Protein structure is inherently a four-dimensional phenomenon, and the dynamic aspects of a protein can be just as important as the mostly static structures typically associated with conventional high-resolution structural biology methods, such as X-ray crystallography. In recent years, gas-phase methods, such as native mass spectrometry and ion mobility spectrometry, have been developed that lack the high resolution of crystallography, nuclear magnetic resonance, or cryo-electron microscopy, but can complement these methods such that the combination of different techniques yields a better understanding of the structural ensemble [1,2,3,4,5,6,7]. A benefit of MS-based methods is their near-universal applicability and relatively high throughput. While it is now generally accepted that the gas-phase structure of proteins in native MS reflects important aspects of the solution structure, the question remains to what extent subtle interactions are preserved [3].

One way to combine the analytical benefits of mass spectrometry with the ability to confidently probe protein structure as it appears in solution is to use chemical labelling [8,9,10]. This can be performed in a way that is sensitive to protein conformation in solution, and subsequent MS analysis allows identification of the modification sites, which facilitates correlation of primary and higher-order structure. Such methods provide valuable complementary information to conventional structural biology, for example, allowing the convenient probing of membrane proteins, or monitoring the response to a thermal or chemical perturbation of the structure on a (sub)millisecond timescale [11,12,13,14,15]. These methods also have the benefit that their experimental feasibility typically only has a limited dependence on protein mass [16].

Arguably, the most common labelling method in use today is hydrogen–deuterium exchange (HDX), which allows modification of backbone amide groups in a way that is sensitive to both solvent accessibility and hydrogen bonding, i.e., secondary structure [17,18,19,20]. While this is a powerful method, the kinetics of the exchange reaction are highly sensitive to experimental factors, such as temperature and pH, and, therefore, considerable expertise is required for a successful HDX experiment, which, as a result, is still hardly a routine approach. Furthermore, analysis is typically performed with enzymatic digestion at low pH and temperature, followed by chromatographic separation and MS analysis of peptides. Due to the reversible nature of the labelling reaction, there is always a degree of back-exchange during this step of the experiment, and this has to be carefully controlled. For this reason, the best results are often obtained using automated sample preparation and handling, which allows better precision and accuracy than human-level control over the experiment. Finally, due to the mobilisation of protons (or deuterons) in gas-phase peptides under high-energy conditions, the most common fragmentation technique in mass spectrometry—collision-induced dissociation—causes randomisation (‘scrambling’) of labelling sites, which limits the resolution of the method to the peptide level [21,22].

For these reasons, irreversible covalent labelling in MS-based structural biology can offer valuable additional information. Different chemistries have been used for this over the years, ranging from conventional substitutions to radical chemistry, for example, with carbenes [10,23,24,25]. Great strides have been made in the use of hydroxyl radicals for footprinting after homolytic dissociation of hydrogen peroxide upon ultraviolet irradiation, typically employing an excimer laser. This method is known as fast photochemical oxidation of proteins (FPOP) [26,27,28]. Due to the very short lifetime of the radicals (particularly as a scavenger is typically added to the solution) it is possible to probe the evolution of protein structure on a microsecond timescale, although it should be noted that Vahidi and Konermann have shown evidence that it can take up to milliseconds for all metastable secondary radicals to be destroyed [29]. The use of hydroxyl radicals is particularly attractive, as they are essentially the same size as water molecules and labelling, therefore, captures the biologically relevant solvent-accessible surface. Before the development of FPOP, Chance and colleagues demonstrated the use of synchrotron radiation to form hydroxyl radicals from water, also leading to selective labelling of the exposed surface of a protein [30,31]. While powerful, these methods rely on access to very specialised equipment and, as such, are out of reach for most researchers. Therefore, there is a need for robust conformation-sensitive labelling approaches that can be easily implemented in more routine settings.

Hydroxyl radicals can of course be conveniently produced in solution in a number of ways, most famously by Fenton chemistry. In this case, hydroxyl radicals are produced by reaction of hydrogen peroxide with Fe(II), yielding Fe(III), OH^-^, and OH^•^, the latter of which can react with amino acid side chains. For a residue-specific overview of possible modifications, we refer the reader to the excellent recent review by Gross and colleagues [10]. Pioneering work by Tullius and Dombrowski demonstrated the use of an elegant Fenton system for probing protein–DNA interactions [32]. In this work, radical production was achieved by a redox cycle involving Fe(II)–EDTA, hydrogen peroxide, and ascorbate to regenerate the Fe(II). Attack by the hydroxyl radical then led to cleavage of exposed parts of the DNA backbone. Subsequent analysis of DNA fragments was performed by gel electrophoresis; however, possible modifications to the protein interaction partner were largely ignored. Interestingly, despite continued use of Fenton chemistry for characterising the structure of nucleic acids bound to protein, there has been limited interest in its use for oxidative footprinting of proteins compared to radiolysis [10,33,34,35,36]. Several reasons have been reported for this; for example, (i) the Fenton reaction cannot be initiated and quenched on the same timescale as FPOP; (ii) there is the risk that iron, EDTA, or other components in solution might interact with the protein of interest and induce a conformational change; and (iii) letting the process continue for too long could permit undesired secondary reactions [37].

**Figure 1:**
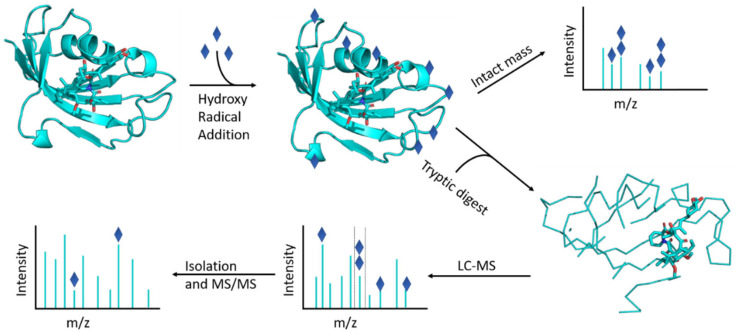
Schematic representation of the oxidative footprinting workflow.

Despite these concerns, here we explored whether Fenton chemistry can be combined with routine proteomics sample preparation and LC-MS to reveal insight into protein structure and dynamics. The overall workflow is represented schematically in Figure 1. Using a set of model proteins with masses between 10 and 150 kDa, we found that we were indeed able to selectively label the exposed protein surface. For the FK1 domain of the immunophilin FKBP51—currently an important drug target due to its relevance in mood disorders [38,39]—differences in the modification pattern between the ligand-free and -bound form were consistent with the known binding site of two different ligands, based on crystallography [40,41]. We were able to apply the same method to complexes of the homologue FKBP12 without any particular difficulty. Finally, in the case of myoglobin, we unexpectedly found that the iron centre in the noncovalently bound haem group was also able to participate in the Fenton reaction. As a result, oxidative footprinting for this protein reflected not only surface exposure in a way consistent with the known structure and labelling experiments from the FPOP literature, but also reflected the binding mode of oxygen to this prosthetic group.

## 2. Results

We used four model proteins to benchmark our method in this study: one large, noncovalent complex in which sequence regions involved in protein–protein binding interfaces constitute a clearly defined protected ‘core’, a smaller protein with a noncovalent haem group, and two small drug targets. Interestingly, each of these model systems highlighted a different aspect of the use of Fenton chemistry for oxidative footprinting, and the presentation of the results is, therefore, organised by protein substrate.

### 2.1. Myoglobin: Intrinsic Fenton Reactivity Reflects Co-Ordination Mode of the Haem Group

**Figure 2:**
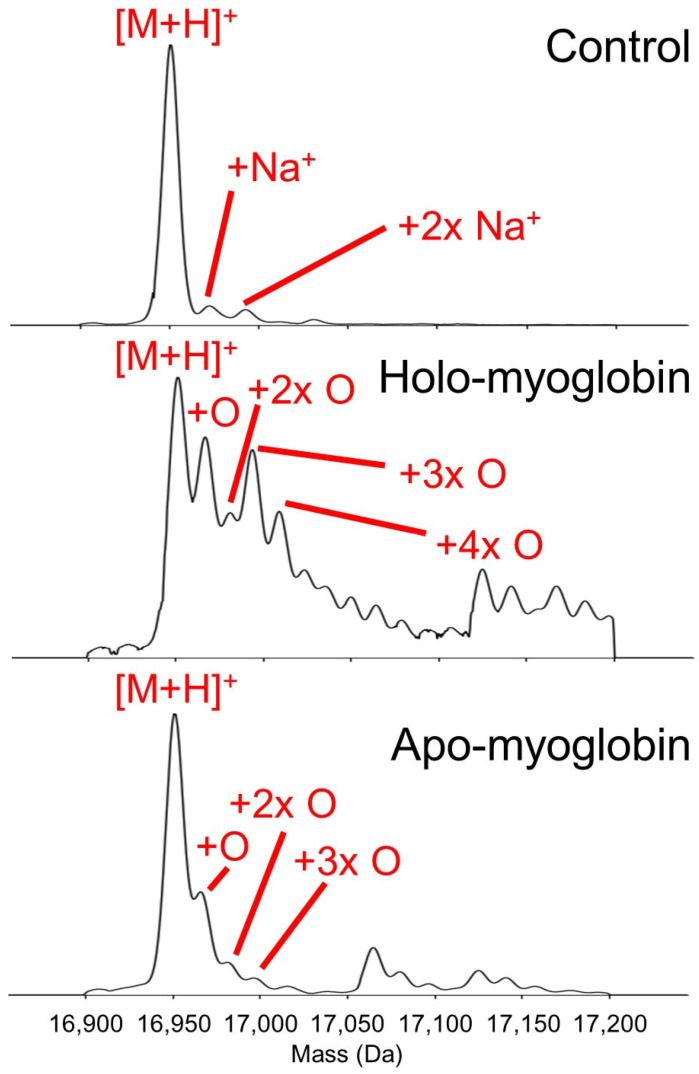
Deconvoluted mass spectra of *apo*-myoglobin and *holo*-myoglobin before (‘control’ experiment in the top panel) or after oxidative footprinting. Oxidative footprinting was carried out under native-like conditions, but samples were chemically denatured prior to MS measurement, which is why the haem group is not retained in *holo*-myoglobin. Only one spectrum is shown in the top panel as *apo*- and *holo*-myoglobin are identical after chemical denaturation.

As a first model system, we decided to use myoglobin, as this 17.6 kDa protein (including a haem group with a mass of 616 Da) has been well characterised by various techniques, including several reports in the early FPOP literature [26,27]. As such, we were eager to determine how the oxidation pattern in Fenton-chemistry-based footprinting would compare to those published results. Furthermore, it is well known that loss of the prosthetic haem group causes significant structural destabilisation in this protein, especially in the C-terminal half of the sequence (helices F, G, and H) [26,27,42]. Therefore, we anticipated that comparing results for *holo*- and *apo*-myoglobin would provide a clue about the ability of our method to distinguish between different conformational states. After quenching the footprinting reaction, an aliquot was taken for intact mass measurement, which was performed under denaturing conditions. Results are shown in Figure 2. In agreement with the FPOP literature, a fairly similar overall level of oxidation was observed for *apo*- and *holo*-myoglobin, with the latter appearing slightly more oxidation-sensitive [26,27].

**Figure 3:**
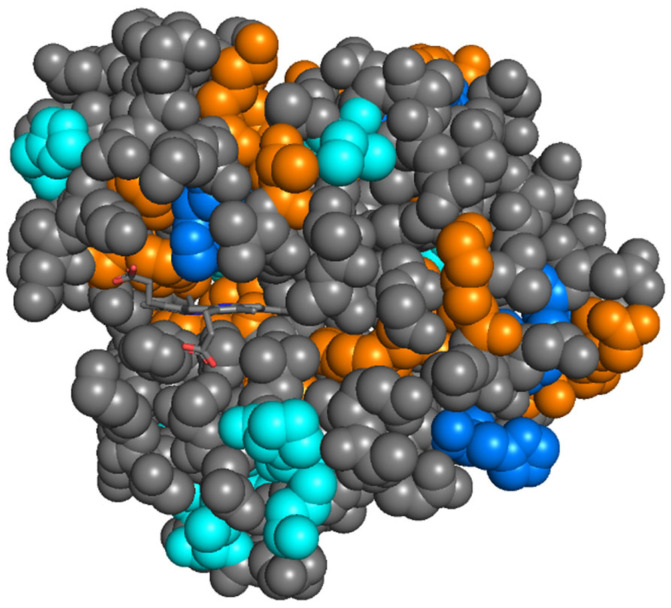
Crystal structure of *holo*-myoglobin (Protein Data Bank accession code 1YMB), with important residues for oxidative footprinting highlighted. Residues that were oxidised in our work that were previously identified as oxidation-sensitive in the FPOP literature are coloured blue. New modifications identified in our work (mostly near the haem group) are coloured orange. Modification sites that were identified in the FPOP literature but not in our experiments are coloured cyan.

Subsequently, oxidation site determination was performed through bottom-up analysis, where differences between both states of the protein were observed. Overall, our data were consistent with those in the early FPOP work, where a good agreement with the exposed surface was already established based on the known crystal structure (see Figure 3) [26,27]. Importantly, as described by Gross and colleagues, His93, which directly co-ordinates the iron centre in the haem group, was efficiently oxidised in *apo*-myoglobin, but was protected from oxidation in *holo*-myoglobin [27]. The main discrepancy between our results and those in previous reports was significant oxidation in *holo*-myoglobin of residues Leu32, Lys42, Phe43, Val68, Leu72, Ile99, Tyr103, and Phe138 in the binding pocket of the haem group (coloured orange in Figure 3). Note that, as described in Materials and Methods, oxidised residues were identified using tandem MS—for example, fragments *b_10_*, *b_11_*, *y_4_*, and *y_6_* from the oxidised peptide L(32)FTGHPETLEKFDK(45) bracketed the Lys42 and Phe43 residues, allowing us to say that these were both oxidised, rather than the mass shift being a result of, for example, double oxidation of phenylalanine.

As we used the same commercial supplier as Gross and colleagues, and the control experiment shown in Figure 2 confirmed the molecular mass and lack of modifications, we assumed the aforementioned discrepancy was due to a difference in reaction conditions rather than a difference in protein structure. Given the different method for generating hydroxyl radicals (flash photolysis vs. Fe/ascorbate-driven redox chemistry), we hypothesised that the iron centre within the haem group was involved in Fenton-like redox cycling in the presence of ascorbate, and that radicals were generated in close proximity to the haem group in this process. To test this, we repeated the experiment, but did not add any extrinsic iron in the form of Fe(II)–EDTA to the reaction mixture (i.e., only ascorbate and hydrogen peroxide). As before, the reaction was quenched after 15 s.

**Figure 4:**
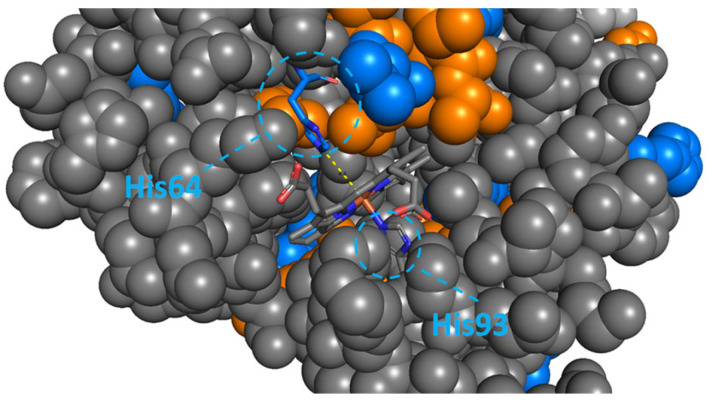
Crystal structure of *holo*-myoglobin, focussed on the binding pocket of the haem group. Highlighted residues were oxidised in the presence of ascorbic acid and hydrogen peroxide, without addition of Fe(II–EDTA. Residues in blue were identified in two independent ways with MaxQuant (either through fragmentation of two overlapping peptides, or from two different oxidative chemical modifications in the same peptide) [44,45]; those in orange were detected once (see Section 4 for details).

Under these conditions, we observed very little oxidation at surface regions remote from the haem group, while residues near this group were, again, oxidised extensively, in good agreement with our hypothesis. Interestingly, His93 and other residues on its side of the plane of the haem group were largely protected, and oxidation was mainly observed in residues located on the opposite side in the native structure, including His64, which also co-ordinates the iron centre. This opposite side is where oxygen binds to iron, displacing His64 in the process (see Figure 4); hence, this oxidation pattern seems to reflect the increased local conformational flexibility and space required to allow the protein to perform its oxygen transport function in vivo. Metal-catalysed oxidation of amino acid residues in the vicinity of biologically relevant metal ions has been observed before [43]; however, here, the oxidation pattern not only provided information about the binding region, but also reflected the solution-phase dynamics of ligand binding to and release from the metal.

### 2.2. ADH: Highly Reactive Sulphur-Containing Side Chains and Surface-Selective Labelling

While our results for myoglobin—including the redox activity of the haem group—were intriguing, we wanted to further investigate the ability of our Fenton-chemistry-based method to selectively modify the solvent-exposed surface of a protein. For this reason, we wanted to use a model system without any redox-active metals. We selected alcohol dehydrogenase (ADH), which forms a 148 kDa tetramer in solution. Bottom-up analysis is not particularly limited by protein mass, and due to the large size of ADH, there is a clear ‘core’ region which is effectively shielded from the solvent. ADH contains two Zn^2+^ ions per monomer (so eight in total for the tetramer); however, this does not interfere with the labelling reaction, as Zn is redox-inactive under these conditions.

**Figure 5:**
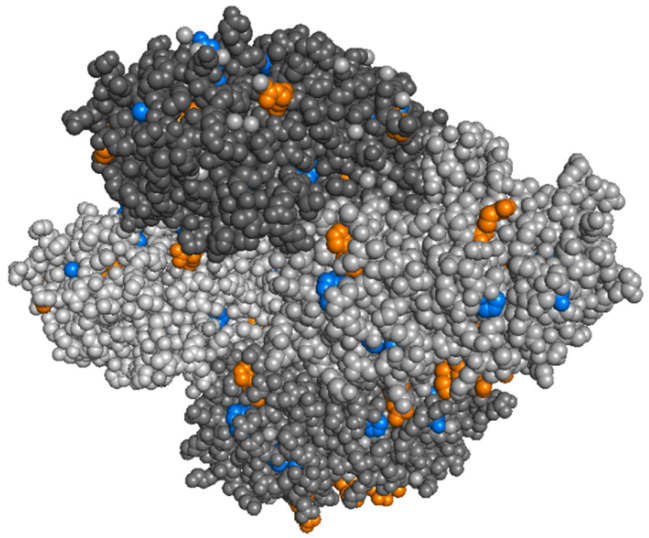
Labelling sites in ADH, indicated in the crystal structure (PDB accession code: 4W6Z) with the same colour code as in Figure 4.

Overall, less extensive modification was observed in ADH compared to myoglobin, possibly reflecting the larger size of the tetramer (see Figure 5). In total, eleven modification sites were identified (note that each chain comprises 347 residues). Comparing the identified modification sites to the accessible surface based on the crystal structure [46], most of the oxidation sites were indeed solvent-exposed, which supports our hypothesis of selective labelling without major structure disruption on the timescale we used. Two exceptions, for which oxidation was observed despite the residues not being classified as accessible, were Met270 and Cys277. For Met270, we found that significant oxidation occurred even during the analysis of a sample where no oxidative footprinting was performed, indicating that this was likely an artefact that occurred on the peptide level during sample preparation or the electrospray process.

In contrast, Cys277 was not oxidised in a control sample that was not exposed to hydroxyl radicals, indicating that this was indeed a result of the footprinting reaction. This was surprising, as our calculations based on the crystal structure indicated that this residue was not solvent-accessible; however, it should be noted that this residue is located in a cleft facing the solvent, rather than being involved in the protein–protein interface. The nearby residues Ala272 and Gly273 are classified as solvent-accessible in the crystal structure, and the residues in this region have relatively high crystallographic B-factors in the 38–46 Å^2^ range, indicating significant local conformational flexibility. As such, it is plausible that Cys277 occasionally comes into contact with the solvent during the normal ‘breathing’ of the protein structure and, given the high intrinsic reactivity of cysteine toward hydroxyl radicals, this could account for the oxidation of this residue that we observed. In this way, it is plausible that our method indirectly provides insights into transient states that are normally ‘invisible’. Overall, our results for ADH support the notion that oxidative labelling under the conditions used by us, indeed, selectively modifies the solvent-accessible surface.

### 2.3. FKBP51 and FKBP12: Key Interactions Drive Remarkable Structural Stabilisation

**Figure 6:**
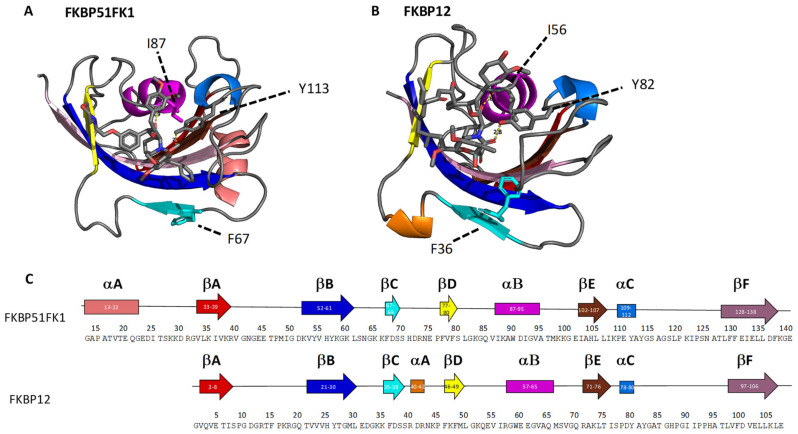
(**A**) Crystal structure of FKBP51FK1 in complex with the SAFit analogue iFit4 (Protein Data Bank accession code 4TW7), with key interacting amino acid residues labelled with a dashed line. (**B**) Crystal structure of FKBP12 in complex with FK506 (accession code 1FKJ), with key interacting amino acid residues labelled with a dashed line. (**C**) Sequence overview for FKBP51FK1 and FKBP12, where homologous sequence regions are aligned. Secondary structure elements are colour-coded and correspond to the same colour in the crystal structures.

The immunophilin FKBP51 (see Figure 6A) belongs to the class of FK506-binding proteins and is a potentially important drug target in the context of depression, obesity-related diabetes, and chronic pain [38,39]. Drug development is hindered by the presence of homologues in the human body, including FKBP52 and FKBP12, which poses a challenge for designing selective ligands that avoid off-target effects [40]. One of the most promising ways for selective inhibition of FKBP51 is the targeting of minor conformational states, which exhibit a greater structural difference between homologues than the most abundant conformation [47]. Understanding such differences in the dynamics of protein structure and identifying possible transient binding sites is a major challenge for structural biology and difficult to achieve with conventional methods [48]. Given the importance of this protein family for human health, we decided to investigate two homologues with our footprinting method—FKBP12 (11.8 kDa, Figure 6B) and FKBP51. For the latter, rather than the full-length protein, a 14.0 kDa construct was used consisting of the FKBP-type peptidyl-prolyl cis-trans isomerase (PPIase) domain (called the FK1 domain), and this construct will be referred to as FKBP51FK1 in the rest of this work. Both proteins were analysed in their free state, as well as bound to two different ligands: SAFit1 and FK[4.3.1]-16h [40,41]. Binding affinity (K_i_) to FKBP12 is approximately 163 nM for SAFit1 and 1.8 nM for FK[4.3.1]-16h. Binding affinity to FKBP51 is approximately 4 nM for SAFit1 and 57 nM for FK[4.3.1]-16h [41,49].

In our initial experiments with the FK506-binding proteins, we found that reproducibility between replicate experiments was somewhat poor, leading to wide confidence intervals on the degree of oxidation, particularly for the complex with FK[4.3.1]-16h (data not shown). Manual inspection of the spectra revealed elution of a significant amount of intact protein near the end of the LC gradient in samples where ligand was present, indicating incomplete digestion. We hypothesised that this was due to the protein structure being sufficiently stabilised by interactions with the ligand to resist unfolding under our standard denaturing conditions (incubation with 6 M urea at 28 °C for one hour). This apparently led to inefficient digestion of largely folded protein by trypsin, somewhat similar to a limited proteolysis experiment [50,51].

Given the suboptimal reproducibility in these initial experiments, we discarded the results from bottom-up analysis and repeated the experiments with more aggressive denaturation conditions (vide infra); however, we did wish to further test the hypothesis of ligand-induced stabilisation toward chemical denaturation. For this, we performed MS of intact FKBP12 in the presence of FK[4.3.1]-16h (the sample that showed the highest abundance of remaining intact protein after digestion) with different concentrations of acetonitrile (data not shown). Under native-like conditions with no organic solvent, the protein was mostly in its ligand-bound form and was observed at low charge states, as commonly observed in native MS. Intriguingly, we found that a significant amount of low-charge-state (likely compact) protein was observed until 45% acetonitrile was added, and even a non-negligible amount of protein–ligand complex was still present under these conditions. In contrast, for the ligand-free FKBP12, a ‘steady-state’ of mostly high-charge-state (likely unfolded) protein was observed at 35% organic solvent, and this did not change until >50%, at which point precipitation of the protein occurred. This observation supports the notion that ligand binding stabilised the protein toward denaturation and subsequent enzymatic digestion. For comparison, the presence of 30% acetonitrile was sufficient to cause myoglobin to mostly lose its haem group. To avoid incomplete protein digestion and ensure reproducibility, the experiments were repeated, with the denaturation step being extended to six hours. Three independent samples were prepared for each condition (two proteins, each in their free state and bound to both ligands), and each sample was injected onto the column twice (i.e., a total of 36 injections were performed).

**Figure 7:**
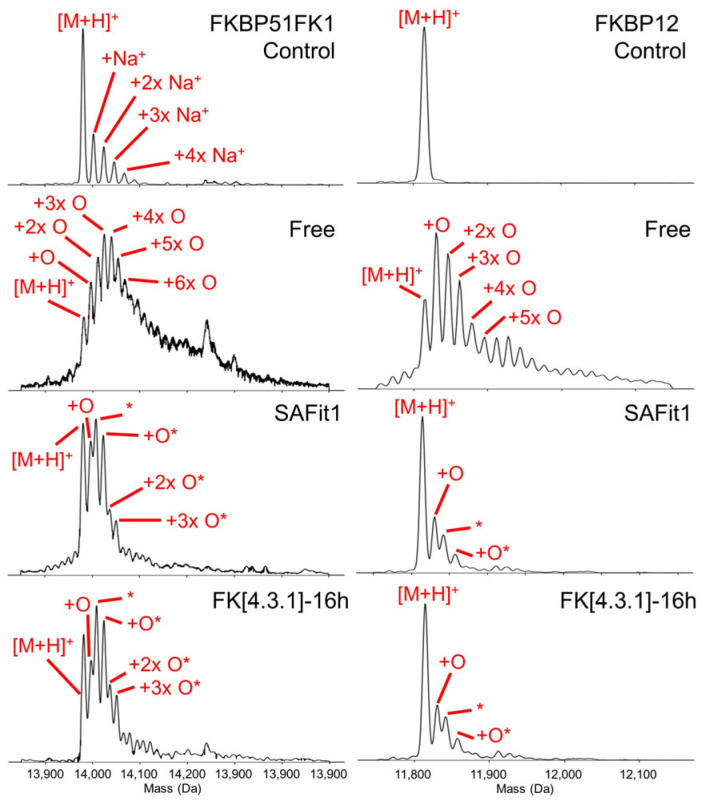
Intact mass measurement of FKBP51FK1 and FKBP12 (deconvoluted spectra shown) under denaturing conditions before (control; top panel) and after oxidative footprinting, either in the absence (second row; ‘free’ protein) or presence of ligands SAFit1 and FK[4.3.1]-16h. Peaks labelled with an asterisk carry an additional modification of 27.01 Da.

As before, aliquots were taken and intact mass measurements performed immediately after the oxidative footprinting reaction (Figure 7). This revealed strongly reduced reactivity toward oxidation upon ligand binding, consistent with ligand-induced protection. In addition to the insight into the global labelling extent, a further benefit of this intact mass measurement was that it demonstrated that reaction between FKBP12 and hydroxyl radicals mostly occurred through ‘simple’ oxidation rather than side reactions that have been reported in the literature [10]. This was revealed through the observation of a pattern of mass increases in steps of 16 Da, up to an addition of 48 Da (more extensively oxidised protein was visible in the spectrum, but at lower abundance). With this knowledge, we were able to significantly speed up our data analysis (a necessity, given the sizeable data set) by focussing on peptides with these modifications. In practice, addition of a single oxygen atom was by far the most common modification at the peptide level, which is consistent with global addition of only a few oxygen atoms to the entire protein.

**Figure 8:**
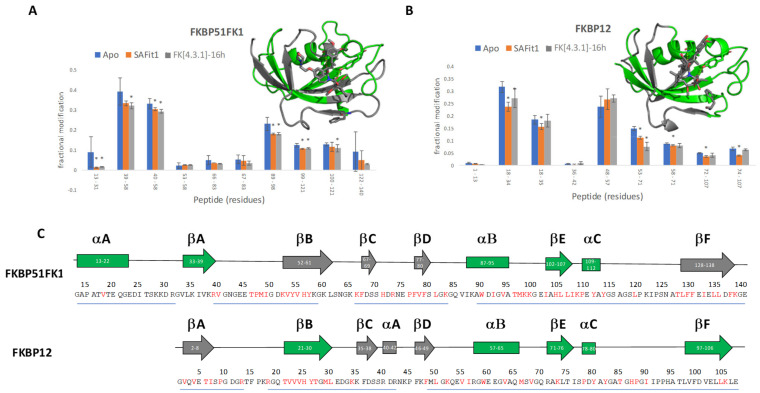
Results of the peptide-level analysis of the FK506-binding proteins: (**A**) fractional oxidative modification of the peptides of FKBP51FK1 for the *apo* protein as well as SAFit1- and FK[4.3.1]-16h-bound protein, in which statistically significant (*p* < 0.05) differences between ligand-bound and -free states are indicated with an asterisk (*). Insets show crystal structures with regions that show reduced oxidation after binding of either ligand in green. (**B**) Fractional modification of the peptides of FKBP12 for the *apo* protein, as well as ligand-bound states. (**C**) Sequence overview for FKBP51FK1 and FKBP12, with sequence regions covered by the observed peptides underlined. Secondary structure elements are labelled as in Figure 6. Elements that show reduced oxidation after ligand binding are coloured green in Panel (**C**), and unaffected elements are in grey (same colour code as the insets in Panel (**A**); no regions were observed where ligand binding led to increased oxidative labelling). Detected oxidative labelling sites are coloured red in the sequence.

Peptides that were detected with sufficient signal-to-noise for quantification covered 92% of the sequence of FKBP12 and 88% of the sequence of FKBP51FK1. Oxidation sites were identified qualitatively with single-residue specificity through tandem MS; however, signal-to-noise in fragment spectra was insufficient to determine site-specific changes in oxidation level with statistical significance; therefore, quantitative analysis was limited to the peptide level. Results of this analysis are summarised in Figure 8. The fact that three samples were prepared for each condition allowed us to evaluate the reproducibility of our method in this case. Visually, it is apparent that most error bars are small; more quantitatively, the median coefficient of variation for the fraction of oxidised peptides for FKBP51FK1 was 10.2%. A similar value (10.5%) was observed for FKBP12 peptides.

The first thing that stands out from these results is the overall reduction in degree of oxidation upon ligand binding for both proteins. This is consistent with the intact mass measurements and could be partly due to direct shielding of reactive residues by the ligands, but also supports the hypothesis that overall structural compaction led to the incomplete digestion of ligand-bound protein that we observed in our initial attempts. Analysing the results in more detail, some interesting differences between both ligands, and between both proteins become apparent. In this discussion, the homology between both proteins is important; specifically, it should be noted that residues 1–106 of FKBP12 show a striking similarity to residues 32–137 of FKBP51FK1. Despite this, even in the ligand-free form, some differences are apparent. Most striking is the very limited degree of oxidation in peptides spanning residues 53–83 in FKBP51FK1, corresponding to the region 22–52 in FKBP12, where significant oxidation was observed. We attribute this to the presence of the N-terminal extension of 31 residues in FKBP51FK1, which appears to shield part of the main β-sheet region of the protein from the solvent in the crystal structure (see Figure 6A). Perhaps due to this limited initial degree of oxidation in FKBP51FK1, no statistically significant reduction was observed in this region after ligand binding, while binding of SAFit1, but not FK[4.3.1]-16h, did lead to a significant protective effect in the 18–34 region of FKBP12, possibly reflecting a greater degree of direct shielding by the bulkier SAFit1.

An alternative, more intriguing explanation for this behaviour than a simple steric effect involves the fact that binding of SAFit1 to FKBP51FK1 requires the side chain of residue Phe67 to be displaced, and this alternative conformation is at the core of the ability of this type of ligand to distinguish between homologues [40]. The binding affinity of SAFit1 to FKBP12, where Phe36 is the counterpart to Phe67 in FKBP51FK1 (see Figure 6), is an order of magnitude lower than to FKBP51FK1 [41,49]. Interestingly, in both cases the key phenylalanine residue is part of a β-sheet, with the adjacent strand (labelled as ‘βB’ in Figure 6 and Figure 8) composed of residues 21–30 in FKBP12 and residues 52–61 in FKBP51FK1. In this context, the increased protection observed in the βB region of FKBP12 after binding of SAFit1 compared to FK[4.3.1]-16h (which binds to an *apo*-like conformation), and the lack of such protection in the corresponding region of FKBP51FK1 upon binding of either ligand, could indicate that a more significant structural rearrangement is required for FKBP12 than for FKBP51 to adopt the conformation that can efficiently bind SAFit1. It is plausible that this need to undergo a more significant rearrangement makes the SAFit1-binding conformation less favourable for FKBP12, which might contribute to the previously established lower binding affinity.

The N-terminal extension itself also exhibits significant protection upon ligand binding to FKBP51FK1, as reflected by the decrease in oxidation in the peptide spanning residues 13–33. This was surprising as, in the crystal structure, this region is fairly distant from the ligand binding site. This effect may be a result of an indirect stabilization caused by the adjacent beta strands that are protected upon ligand binding. In both FKBP12 and FKBP51FK1, the C-terminal portion of the protein showed significant protection after ligand binding. This is unsurprising and can largely be attributed to steric effects, as this region contains many residues that are either part of, or close to, the binding site. Of note is the protection of the peptide with residues 89–98 in FKBP51FK1 and, similarly, that with residues 53–71 in FKBP12. These span an α-helix (αB) containing, or being close to, a key interacting amino acid residue (Ile56 in FKBP12 and Ile87 in FKBP51FK1) that forms a strong hydrogen bond with both ligands through its amide nitrogen atom [49,52,53,54]. Furthermore, a very reactive tryptophan (based on both the inherent reactivity of the side chain toward oxidative labelling, and the direct observation, as shown in Figure 8C) that is located within the binding pocket and directly exposed to solvent in the absence of a ligand is located in this region. The shielding of this reactive tryptophan (Trp59 in FKBP12; Trp90 in FKBP51FK1), combined with the strong interaction of the isoleucine with the ligand, causes one of the most significant protections of the protein.

## 3. Discussion

We have shown that oxidative labelling through Fenton chemistry can be employed for structural characterisation of a set of model proteins, with a reaction time of only a few seconds, and that conformational changes are reflected in the modification pattern. For the smaller (11–18 kDa) proteins we studied, extensive oxidation was observed, and it was demonstrated how the oxidation pattern correlated to protein structure, including dynamic aspects. Information was sparser on the 148 kDa ADH tetramer, where fewer oxidation sites were identified. A plausible explanation for this is that the reaction rate was limited by the concentration of Fenton reagents (specifically Fe(II)–EDTA at 94 µM) under these conditions, leading to approximately the same number of oxidation events being distributed over a much larger number of reactive residues, resulting in the observed greater selectivity for highly reactive sites. A possible way to address this in the future and obtain a consistent degree of oxidation across a range of protein masses would be to use a consistent mass-based concentration for proteins in the labelling solution, rather than consistent molarity.

For the ADH tetramer, we showed that oxidation occurs primarily at the exposed protein surface, in agreement with other hydroxyl radical footprinting techniques. In the cases of myoglobin and the two FK506-binding proteins we tested, there were clear differences in the oxidation pattern between the ligand-bound and ligand-free state of the protein. For myoglobin specifically, a key histidine residue that binds to the native iron centre was highly reactive in the *apo* state, and protected in the *holo* state. Furthermore, oxidation was observed of residues within the binding pocket of the haem group, but exclusively on the side of the plane of this prosthetic group at which biologically relevant ligands, such as oxygen, bind. It is reasonable to assume that this reflects increased conformational flexibility on this side of the haem group, which is necessary to accommodate the exchange and transport of gas molecules by myoglobin. This supports the hypothesis that our method is able to inform on dynamic aspects of protein conformation, rather than just a static lowest-energy structure.

In addition to these strengths, we also identified several practical limitations to the method presented in this work. The reaction time for our oxidative footprinting method is limited in practice to several seconds, which leads to a degree of ensemble averaging and precludes the probing of protein structure on a microsecond timescale, as is possible in labelling methods based on photolysis. The use of microfluidics in future studies could significantly reduce the reaction time [55,56], but—even assuming the extent of the labelling reaction on such a short timescale would be sufficient to obtain structural information—this approach would still be orders of magnitude slower than FPOP.

Another potential concern is the effect of sulphur-containing residues. While this did not pose an issue for most methionine- or cysteine-containing peptides in our hands, we did find that, in the case of ADH, the residue Met270 was consistently and spontaneously oxidised—possibly during sample handling or the electrospray process—even in control samples. This needs to be carefully controlled and could pose a challenge for the analysis of methionine- or cysteine-rich proteins by oxidative footprinting, regardless of the exact chemistry used to generate hydroxyl radicals. The main bottleneck we identified in implementing this type of experiment was data analysis. Given that most amino acid residues are at least somewhat reactive toward hydroxyl radicals, and that many residue types are able to undergo several competing reactions under these conditions, the number of (modified) peptides that need to be matched to an experimental data set, even for a known protein sequence, quickly becomes very large. Even using a high-end desktop PC, searching for all possible reaction products is not feasible, or is at least sufficiently time-consuming to be impractical, and an optimal trade-off between ‘complete’ data analysis and processing time needs to be determined empirically in the absence of access to high-performance computing. For quantitative analysis of oxidised peptides from FK506-binding proteins, we found that a targeted software package (pepFoot) provided good performance while requiring far less computational power than MaxQuant [44,45,57]. In future work, we will further optimise the data processing workflow, as well as extend the method to a greater set of protein–ligand systems, and compare it to other, more conventional labelling techniques.

Combining bottom-up proteomics analysis with intact mass measurement and/or top-down fragmentation can be helpful for optimising the analytical workflow, as it provides a clue regarding the overall extent of modification and possibly some of the labelling sites, which can inform the subsequent more in-depth bottom-up data analysis. Similarly, especially when studying ligand binding, native mass spectrometry can provide important complementary insight to oxidative footprinting. Finally, incomplete protein digestion complicates the data analysis and potentially leads to poor reproducibility, but, at the same time, can be indicative of high structural stability, similar to limited proteolysis approaches developed in recent years. Combining the insights from all the aforementioned data points—and with insights from conventional structural biology methods—leads to an improved understanding of the ‘four-dimensional’ structure of a protein in solution. We believe that the underexplored labelling method used in this work shows sufficient promise to be further developed in the future as a technique for hydroxyl radical footprinting with low barriers to entry compared to radiolysis. As such, this will potentially provide a valuable addition to the toolset of researchers interested in MS-based conformational protein analysis.

## 4. Materials and Methods

### 4.1. Proteins, Reagents and Solvents

Most materials were acquired from commercial suppliers: HPLC-grade acetonitrile (Roth, Karlsruhe, Germany, HN44.2); LC-MS grade acetonitrile (Supelco LiChrosolv, Darmstadt, Germany, 1.00029.2500); alcohol dehydrogenase (Sigma-Aldrich, St. Louis, MI, USA, A3263); ammonia, 30% *w*/*w* (Sigma-Aldrich, St. Louis, MI, USA, 221228); ammonium acetate, 7.5 M (Sigma-Aldrich, St. Louis, MI, USA, A2706); *apo*-myoglobin (Sigma-Aldrich, St. Louis, MI, USA, A8673); L-ascorbic acid (Sigma-Aldrich, St. Louis, MI, USA, 255564); Discovery^®^ DSC-18 SPE Tubes (Sigma-Aldrich, St. Louis, MI, USA, 62602-U); 1,4-dithiothreitol (Roth, Karlsruhe, Germany, 6909.1); H_4_EDTA (Sigma-Aldrich, St. Louis, MI, USA, 431788); formic acid (Fisher Chemical, Waltham, MA, USA, A117-50); *holo*-myoglobin (Sigma-Aldrich, St. Louis, MI, USA, M0630); hydrogen peroxide, 30% *w*/*w* (Sigma-Aldrich, St. Louis, MI, USA, 95321); iodoacetamide (Sigma-Aldrich, St. Louis, MI, USA, I6125); iron(II)-chloride tetrahydrate (Sigma-Aldrich, St. Louis, MI, USA, 44939); Pierce^TM^ Trypsin Protease (Thermo Fisher, Waltham, MA, USA, 90058); triethylammonium bicarbonate buffer (Fluka, St. Louis, MI, USA, 17902); trifluoroacetic acid (Roth, Karlsruhe, Germany, P088.3); urea (Roth, Karlsruhe, Germany, 2317.1). FK506-binding proteins were expressed based on previously described methods [58,59].

### 4.2. Oxidative Footprinting

Protein stock solutions of FKBP12 and FKBP51FK1 in 20 mM HEPES, pH 8.5, and 150 mM NaCl were diluted in 200 mM ammonium acetate to 40.7 µM. Ligand stock solutions were prepared in DMSO at 250 times the final concentration and prediluted to 50 times the final concentration in acetonitrile. A total of 49.2 µL of the protein solution and 0.8 µL of the ligand solution or 200 mM ammonium acetate were mixed to achieve a final protein concentration of 40 µM and ligand concentration of 80 µM, and incubated for 15 min at room temperature, after which the oxidative footprinting reaction was initiated.

For each reaction, fresh solutions were prepared of 0.3 M hydrogen peroxide, 37.5 mM L-ascorbic acid, 187.5 mM thiourea, and iron(II)–EDTA solution. The 0.3 M hydrogen peroxide solution was prepared by diluting a 30% *w*/*w* stock solution with milliQ water. The ascorbic acid solution was prepared by dissolving 6.6 mg of ascorbic acid in 1 mL of milliQ water and neutralising with 2.3 µL of 30% *w*/*w* ammonia. Note that ascorbic acid is oxidation-sensitive in air and that this solution was stable for approximately one hour at room temperature. For a 187.5 mM thiourea solution, we dissolved 14.27 mg of thiourea in 1 mL of 200 mM ammonium acetate solution. The iron(II)–EDTA solution was prepared using a stock solution of 3 mM H_4_EDTA with 12 mM ammonia. A 1.5 mM iron(II)-chloride solution in milliQ water was made fresh for the reaction. Equal volumes of the EDTA stock solution and the iron(II)-chloride solution were mixed in a reaction tube to obtain the iron(II)–EDTA solution.

For the oxidative footprinting reaction, 50 µL of a 40 µM protein solution (in 200 mM ammonium acetate) was pipetted into a 500 µL reaction tube. Next, 10 µL of the iron(II)–EDTA solution was added, followed by 10 µL of the L-ascorbic acid solution. Immediately after subsequently adding 10 µL of the 0.3 M hydrogen peroxide solution, the reaction tube was vortexed and the reaction was allowed to proceed for 15 s. After 15 s, the reaction was quenched by adding 20 µL of the thiourea solution into the reaction tube and vortexing. The final concentration of the protein after the reaction was 5 to 20 µM with 140 mM ammonium acetate.

### 4.3. Tryptic Digest

For digestion, 300 µL of an 8 M urea solution in 50 mM TEAB, pH 8.5, with 100 mM NaCl, as well as 8 µL of a 0.5 M solution of DTT were added to the oxidative footprinting reaction mixture. After incubating the mixture for one hour at 28 °C, 1 mL of 50 mM TEAB, pH 8.5, and 1.2 µL of 1 mg/mL trypsin in 50 mM acetic acid were added in succession. The digest was incubated overnight at 37 °C. After the samples cooled down to room temperature, trifluoroacetic acid was added until the solution was at a pH value of 2. A 100 mg C18-SPE cartridge was conditioned with 1 mL HPLC-grade acetonitrile and 1 mL 0.6% *v*/*v* TFA solution with milliQ water. Next, the sample was loaded on the cartridge and washed with 1 mL 0.6% *v*/*v* TFA solution in milliQ water. Elution was performed with 1 mL of an 80% *v*/*v* acetonitrile solution. The eluate was dried in a vacuum centrifuge (UniVapo 150H; UniEquip, Planegg, Germany) and redissolved in 100 µL of a 5% *v*/*v* acetonitrile solution containing 0.1% *v*/*v* formic acid.

### 4.4. LC-MS/MS Analysis

LC-MS/MS analysis was performed with an LTQ Orbitrap XL (Thermo Fisher Scientific, Waltham, MA, USA) controlled by Xcalibur 2.1 and a micro-LC system consisting of a Micro Pro syringe pump (Eldex Laboratories, Napa, CA, USA), and an Endurance autosampler (Spark Holland, Emmen, The Netherlands) controlled by the Endurance software. Acquisitions were started upon injection by contact closure.

Samples (5 µL) were injected with a flushed loop injection and peptides were separated on a ZORBAX StableBond C18, 0.3 × 150 mm, 3.5 µm column (Agilent, Santa Clara, CA, USA) at a flow rate of 5 µL/min using the following gradient: linear gradient from 5% B to 60% B in 60 min, 10 min linear gradient to 100% B, 10 min at 100% B isocratic, followed by re-equilibration at 5% B for 15 min, with solvent A being water with 0.1% formic acid and solvent B being acetonitrile with 0.1% formic acid.

The mass spectrometer was operated in a data-dependent mode with a precursor scan in the Orbitrap with a resolution of 60,000 at *m*/*z* 400, followed by fragmentation of peptide ions with a charge state of 2 or higher, giving rise to the four most intense signals in the ion trap using CID with a normalized collision energy of 25. Dynamic exclusion was enabled and set to a repeat count of 2 with a repeat duration of 30 s, the exclusion list size was 200, and the exclusion duration was 50 s. The ESI source was operated with 10 units of sheath gas flow rate, a spray voltage of 4 kV, a capillary temperature of 300 °C, a capillary voltage of 3 V, and tube lens set to 30.

For intact mass measurements and for native MS, including the experiments with different concentrations of acetonitrile, 10 µL of sample was loaded into a glass needle that was pulled to a tip of ca. 1-μm orifice diameter with a P97 Flaming/Brown type micropipette puller (Sutter Instrument Co., Novato, CA, USA), starting from 1.2-mm thin-walled glass capillaries (World Precision Instruments, Friedberg, Germany). Ionisation was then performed using a home-built nano-electrospray source that was coupled to the LTQ Orbitrap XL instrument. Intact protein spectra were deconvoluted with UniDec [60].

### 4.5. Data Analysis Using MaxQuant

The MaxQuant calculations were separated into two parts and, in all cases, a precursor mass accuracy of 4.5 ppm was used. In the first part with one calculation run, the unmodified peptides were identified. Only the fasta file of the target protein was used to search against. The default settings were used, with the following exceptions: no fractions—yes; min. peptide length—5; max. peptide mass (Da)—4800; min. score for modified peptides—0; second peptides—off; unknown MS/MS match tolerance and unit—0.5 Da; unknown MS/MS de novo tolerance and unit—0.25 Da; unknown deisotoping—off. For every identified peptide, a separate fasta file was then created for use in the second step. This step comprised three calculation runs to determine the modifications. The first calculation run included +15.995 Da (+O) for M, C, W, Y, F, K, R, Q, D, T, S, A, E, L, I, K, H, N, V and +31.990 Da (+2xO) for M, C, W, Y, F, both as variable modifications additional to carbamidomethyl- and acetyl-(N-term) modifications. The second calculation run included +15.995 Da (+O) for W, C, M, Y, F, H; +47.985 Da (+3xO) for C, W, Y, F; −43.053 Da (+O -5xH -3xN -C) for R; −32.008 Da (+O -S -4xH -C) for M; −30.011 Da (-2xH -C -O) for E, D; +13,9792645 (+O -2H) for L, I, V, P, R, K, E, Q; and −2.016 Da (-2xH) for T, S, all as variable modifications additional to carbamidomethyl- and acetyl-(N-term) modifications. The last calculation run included +15.995 Da (+O) for W, C, M, Y, F, H; −10.032 Da (+2xO -2xH -2xN -C) for H; −4.979 Da (+2xO -H -C -N) for H; −22.032 Da (+2xO -2xH -2xC -2xN) for H; and −23.016 Da (+O -H -N -2xC) for H, all as variable modifications additional to carbamidomethyl- and acetyl-(N-term) modifications. The default settings were used, with the following exceptions: no fractions—yes; digestion mode—no digestion; include contaminates—off; min. peptide length—5; max. peptide mass (Da)—4800; min. score for modified peptides—0; second peptides—off; unknown MS/MS match tolerance and unit—0.5 Da; unknown MS/MS de novo tolerance and unit—0.25 Da; unknown deisotoping—off. For positive identification of an oxidatively modified residue, while avoiding false positive results, we typically required that a +15.995 Da (+O) modification was detected two times in different modified peptides, or, alternatively, that a product from a side reaction of oxidative footprinting was found in addition to a +15.995 Da (+O) modification. Finally, an additional search was run against the entire UniProt database to ensure that peptides identified as oxidised were genuine and not false positives due to overlap with peptides from protein contaminants (note that such overlap would need to occur at both the MS and MS/MS level and is, therefore, very unlikely). Other than the trypsin used for proteolysis and a low-level contamination of keratin in a handful of samples, no other contaminants were found, which confirms sample purity and rules out false positives.

### 4.6. Quantifying Peptides Using pepFoot

Raw files were converted to the mz5 format using MSConvert and then processed in the pepFoot software [23,24,57] using the following parameters: modifications—carbamidomethyl, variable modifications—oxidation (+ oxygen), digestion—trypsin, peptide length—5–40, peptide charge—1–6, # missed cleavages—2, MS tolerance—20 ppm. Extracted ion chromatograms of identified peptides by MaxQuant in acceptable abundance (S/N > 9:1) were then integrated for the modified and unmodified peptide, and the degree of modification was calculated by the software for *apo* and *holo* proteins.

## Data Availability

The data and materials underlying this article will be shared on request to the corresponding author.
